# Medical Association of Central New York

**Published:** 1908-04

**Authors:** C. A. Greenleaf


### SOCIETY PROCEEDINGS.

### Medical Association of Central New York

Reported by C. A. GREENLEAF, M. D., Secretary

Edited by WILLIAM C. KRAUSS, M. D.

THE fortieth annual meeting of the Medical Association of Central New York, was held on October 15, 1907, at the Genesee Valley Club, Rochester, N. Y., and the morning session was called to order at 10.25 A. M., by the president, Dr. W. B. Jones of Rochester.

It was moved and seconded that the reading of the minutes of the last meeting be omitted and that they be approved as printed. Carried.

The report of the treasurer, Dr. Wm. M. Brown, was presented and on motion made, seconded and carried.

With Dr. Chas. A. Vander Beek in the chair, the president, Dr. W. B. Jones delivered his address. Published in the January number of the Journal.

Dr. Henry L. Elsner of Syracuse, read a paper entitled: Two Clinical Reports; (*a*) A Symmetrical Hyperostosis; (*b*) Spinal Hemorrhage.

DISCUSSION.

Dr. A. McPhedran of Toronto, Canada: Although I have been much interested in the presentation of these clinical reports by Dr. Elsner, I will not take up your time in their discussion for the reason that I have not for some time done any special work along these lines. I may say, however, concerning the second case, that we cannot clear such cases up too quickly. It may have been, it seems to me, one of infection. Was there not some disturbance of temperature pointing to acute infection? The loss of sphincter control is to be accounted for either by pressure or destruction of cord substance.

Dr. C. A. Vander Beek of Rochester: I should like to call attention to a case similar to the first described by Dr. Elsner. She was an old woman who was frequently to be seen in the streets of Rochester some years ago. My attention was called to the case by our president, Dr. W. B. Jones, and I subsequently succeeded in obtaining some excellent photographs and, finally, an autopsy. The woman was 62 years old and had had three children. A diagnosis of hydrocephalus had been made by one of our older physicians (since dead) and this diagnosis had stood unchallanged for twenty years. The physicians who had made and acquiesced in this diagnosis had failed to recognise the important fact that the muscles of the neck were small and that the movements of the head were made quickly and with apparent ease. This was one of the first points which aroused my suspicions as to the accuracy of the former opinions. The head was not exactly symmetrical, there were bony ridges on both sides, but they were more pronounced on one side. The facial bones were not enlarged—only the cranial. A superficial post mortem showed no enlargement of the brain cavity, but the thickening of the skull was remarkable. Microscopically there was nothing wrong with the brain.

The reflexes in this patient were perfect, but her vision was not good and she had been deaf for years. These sense impairments together with her disfigurement lead naturally to a mental isolation with a resulting paucity of ideas.

I removed a section from the posterior skull and it measured two inches in thickness. The temporal thickness was an inch and a quarter. This increased thickness was due entirely to the cancellous tissue between the tables, these latter being exceedingly thin.

Dr. E. B. Angell of Rochester: I should like to say a few words emphasising certain features of cases like the second reported by Dr. Elsner. In traumatic neuroses the general practitioner does not seem to realise how severe an injury it takes to produce actual destruction of the spinal cord. The diagnosis of hemorrhage would be enough without regard to whether the hemorrhage was within or around the cord. I am rather inclined to hesitate to accept such diagnosis in this case, on account of the promptness of the recovery. There ought to be an excessive knee jerk in these cases although this is not important.

Dr. M. M. Lucid of Cortland: I had a case similar to the last one, which was presumably one of hemorrhage of the cord. Although Dr. Elsner's case was of only a few months duration, mine extended over a period of two years. There is certainly reason for Dr. Elsner's suggestion as to the ignorance with which many of these cases are handled. In my case there was a complicating circumstance of a $5,000 claim.

Dr. H. L. Elsner of Syracuse: It undoubtedly makes a great difference with the prognosis whether there is the question of settlement by a corporation or whether there is no legal action pending, that is if there are no true organic changes. Dr. Lucid's case was supposed to be a fracture of the spine from a fall on a side-walk. You can locate the spinal lesion and then when the patient sues for $20,000 or $30,000, be sure whether or no you have extra symptoms which do not fit in. We were put on the stand against a man who had studied his case well, in general, but who had no symptoms justifying a localisation in the region where the supposed disease was situated.

I entirely agree with Dr. Angell that it makes no difference whether the hemorrhage was in or around the cord.

In my case there was no question of a money settlement involved. This is, indeed, a broad subject, but let us hold fast to one fact—we must study the functions of the cord, and this can be done with the aid of electricity.

In answer to Dr. McPhedron's suggestion as to the possibility of infection as an etiological consideration, I would state that there were no symptoms of infection present.

I would call attention to the fact that in the case described by Dr. Van der Beek, the vision and hearing were affected, this is usual in these cases. Furthermore, the changes in the bony structure are, as noted by him, usually between the tables. In some cases cysts form in the bone. We will keep an eye on this woman and hope that we may be able to give a microscopical picture of the structural changes.

An Intraperitoneal Method for the Radical Cure of Abdominal Hernias, by M. M. Lucid, Cortland, was then read.

DISCUSSION.

DR. JAMES H. LEARY of Rush: This is certainly a very pretty operation. One difficulty suggests itself to me in rotation to the operation as described by Dr. Lucid. How is it possible to bring the flap out from the ring after it is formed? After the peritoneum is passed out, how is one to pull back the sac once passed out, and it must be dissected out.

DR. M. M. LUCID of Cortland: The operation applies to small hernias and those which are reducible and in which the sac is free or with adhesions, which are usually slight and may be easily broken up.

The Status of Surgical Treatment of General Septic Peritonitis, by J. Wesley Bovee, Washington, D. C. Published in February number of the JOURNAL.

DISCUSSION.

DR. PARKER SYMS of New York City: I have been greatly interested in Dr. Bovee's able treatment of this important subject. A few years ago, I presented a report of all the cases of appendicitis treated in Lebanon Hospital. The rule there was to operate unless the patient was absolutely moribund. My idea in going into this investigation was to find out the opinion on the Ochsner treatment. This seemed to me irrational. His plan was absolute suspension of food in the stomach, rest and rectal enema—and he reported excellent results. He operates after the attack. If they recover, then operate. The consensus of opinion, however, was to operate *at once* on all cases. My own experience agrees almost entirely with that of Dr. Bovee. To my mind the important points are to operate rapidly, to remove the organ when practicable and drain. The after treatment is essentially that of Ochsner—keeping the stomach at rest for forty-eight hours, rectal feeding and saline injections or seeping in. Except in certain circumstances we use the Fowler posture.

DR. W. B. JOHNSON of Batavia: I have been present at many discussions of peritonitis in the past ten years and have come away with various opinions. I was at Saratoga in 1902. I came home, had two cases and lost them both. Had sixty before that. Then in the next six I lost two. Then I went to Toronto and came home with the "let alone" treatment—and lost the first case. What we must have in these cases is a differential diagnosis from the bacteriological point of view—and we want it before the post mortem, too. We should carry the culture tube to our operations just as we do the scalpel. Another important point is as to how quickly we get in. If we operate late, there is a large dose of toxines and we are unfortunate.

Dr. L. W. Rose of Rochester: I would like to ask Dr. Bovee to outline his method in cases of septic peritonitis in which the avenue of infection is through the vagina and uterus.

Dr. Bovee of Washington, D. C.: I decide according to the peculiarities of each case. If the pus is high, then I enter by the abdominal route as the infection dies out. If the infection is gonorrheal, I usually remove the infected part per vaginam. If the infection is acute, I usually let alone, unless there are urgent symptoms. I have not heard a great deal of the medical treatment, but have seen evidences of a live interest in it.

A Case of Dibotrocephalus Latus Infection with specimen, by Dr. Charles O. Boswell, Rochester. Published in April number of the Journal.

DISCUSSION.

Dr. Charles G. Stockton of Buffalo: I would like to ask the doctor what the degree of eosinophilia was in his case?

Dr. Chas. O. Boswell of Rochester: It was not more than five per cent.

Dr. Charles G. Stockton of Buffalo: I now think that we are not giving enough attention to blood examinations in this class of cases. One family in particular has driven me to look into this matter. The father was under my care for six months and then had an attack of appendicitis, after which he went abroad and heard of Ehrlich. Ehrlich found Lumbracoides. One son had eight ova. Another son has six per cent. of eosins and I am now looking for ova. My attention was called to the case by the appendix. The patient is persuaded that the worms were the cause of the appendicitis, and I am not at all sure that he is wrong. Finally I would emphasise the importance of studying the blood for evidences of parasites.

Dr. A. McPhedran of Toronto, Canada: I have seen two patients under two years who were supposed to have typhoid. There was no leucocytosis—nothing found. A colleague observed the case, diagnosed round worm and gave santonin with relief. The other case, investigated by the house physician had a similar history.

Dr. Chas. O. Boswell of Rochester: I wish to thank the gentlemen for their kindly discussion of my paper. The question of the indefinite effects frequently following in the train of parasites covers a large field. Wherever there is an increase in the percentage of eosinophilia the stools should be examined. In my case the eosins were normal. Whenever we think we have a case of pernicious anemia we should examine the stools.

This class of cases is much more common in American than is generally supposed.

The Opium Treatment of Peritonitis, by Charles G. Stockton, Buffalo. Published in the February number of the JOURNAL.

DISCUSSION.

DR. BOVEE of Washington, D. C.: I wish to thank the society for the opportunity for this exchange of views on so important a subject, but there is little real difference between Dr. Stockton and myself. I mentioned cases similar to those alluded to by him. I do hesitate about giving large doses of opium on account of their stimulating effect on the kidneys. One of the great troubles with these toxemias is the attendant nephritis, and for this reason I have been inclined to consider opium harmful.

DR. CHAS. R. BARBER of Rochester: I was sorry not to have heard the whole presentation of this subject, but I have heard the latter part of the discussion and it has appealed to me as rare good sense. The treatment of peritonitis is a game of finesse and certain cases are only to be treated successfully with opium—otherwise they go on to death. I have had some little experience with these cases and formerly used to favor opening them all, but the results in the main were not encouraging. Then with change of method, I had three cases of septic peritonitis and they all recovered. I lock up the bowels for a week, if necessary.

DR. W. L. WALLACE of Syracuse: In certain stages of some cases it is extremely dangerous to operate—then again, it is dangerous to lock up the bowels. When patients die of septicemic poisoning the result follows from the overloading of the system and kidneys with toxines. If the patient comes a little bit early for successful operation, I give a heroic dose of opium and then operate without anesthetic.

DR. CHARLES G. STOCKTON of Buffalo: I have been greatly pleased that this discussion has taken place because it enables me to combat an opinion concerning the use of opium in peritonitis which seems to me erroneous, and I wish to thank Dr. Bovee for his clear exposition of the subject. In the main we agree but we differ absolutely on one point. Now, what is the effect of abdominal shock on the kidneys, bowels, elimination, etc? The bowels are in a state of spasm and conditions are not favorable for elimination through the kidneys. Under such conditions opium helps rather than hinders elimination. The effect of opium in such conditions is very different from its ordinary effect.

Take salt solution or strychnia, for example—they are ineffective in shock in these cases. You must remove the cause of irritation to the splanchnics. And let me emphasise the fact that I refer in this connection not to the use of opium in small doses nor in moderate doses but in heroic doses. I think that in ordinary doses, the use of opium does more harm than good. If you use opium at all in these cases, give it in the largest safe dose and keep the patient under its influence. I have seen severe cases of septic peritonitis recover on this opium treatment. I may seem unduly enthusiastic in this matter because of the poor success of those who *will not* try opium in these large doses.

Dr. J. Wesley Bovee of Washington, D. C.: I wish to call attention to one point on which I must differ from Dr. Stockton. In my opinion, shock from whatever cause is best treated with salt solution.

The two following papers were read and discussion of both papers followed:

The Early Diagnosis of Gastric Cancer, by A. McPhedran, Toronto, Canada. Published in the April number of the Journal.

Report of a Case of Partial Gastrectomy—Presentation of Patient, by Thomas Jameson, Rochester, N. Y.

DISCUSSION.

Dr. Allen A. Jones of Buffalo: This has indeed been a delightful dissertation and eminently practical, especially as to the presence of hydrochloric acid in carcinoma. I have had a number of these cases in the past few years, and some illustrate the clinical fact that there is a transition from gastric ulcer into carcinoma. Gastric ulcer is certainly a predisposing cause to carcinoma. Some of these cases show an excess of hydrochloric acid with no Oppler-Boas bacilli present. Unless operative measures are employed the hydrochloric acid fails with the development of lactic acid. Dr. McPhedran's case was interesting as an representative case. What we need in these cases is the closest study and then insistance upon operation. What the pathological condition was that induced the lactic acid, Dr. McPhedran did not state, but one would expect to find a number of lactic acid bacilli present.

Dr. Chas. G. Stockton of Buffalo: The admirable paper of Dr. McPhedran's recalls to my mind several like cases of gastric disease in which three or four conditions were present calling for differential diagnosis. The common difficulty in such cases is the lack of frequent examinations of the stomach contents. Each time you make such careful examination you get a little nearer to an accurate diagnosis and finally you are able to say that the case is one of gastritis, or of cancer or whatever the actual condition may be. These final approaches are to be made only by repeated and careful examinations made day after day.

Dr. L. W. Rose of Rochester: Dr. McPhedran has given us a number of good points concerning the early diagnosis of gastric cancer. He also has called the surgeon to account for not making a diagnosis before operating.

When the trained internist with the aid of the modern physiological chemist has been in so many instances unable to give an early positive diagnosis, and when he is present and witnesses the gross pathology shown by an exploratory incision and still hesitates, and furthermore, when the pathological area is removed and submitted to the microscopist, and he also expresses a doubt as to the nature of the lesion—then, it seems to me that the internist should not only welcome but insist upon an early exploratory incision.

Dr. J. E. Walker of Hornell: I think that Dr. Jameson should consider it a subject for congratulation that he was not able to present his patient. When it is stated that in spite of the fact that the tumor was so far advanced at the time of operation, and that the patient is now walking about the streets so that he cannot be found, enough has been said as to the entire success of the operation.

Dr. A. McPhedran of Toronto: I will not further trespass on your time. Everything said has been kind and commendatory. I might say a few words on one point—as to whether the power of digestion is not more important than the amount of hydrochloric acid. I take it that the power of digestion includes both the chemical changes, and the mechanical ability of the stomach to empty itself. If the stomach retains the food, it has not performed its whole function. With regard to the causal relation between gastric ulcer and cancer, I would suggest that every cancer is preceded by something, but I do not believe that so large a proportion are preceded by ulcer. Hyperacidity is extremely common. Why should not these cases develop cancer? There are plenty of abrasions as nuclei for the malignant growth. I wish to emphasise the suggestion of Dr. Stockton, as to the necessity for examinations of the stomach contents, repeated again and again. The patients will not object—they will consent to anything in reason.

Danger of Pregnancy following Operations for Cancer of the Breast, by William S. Cheesman, Auburn. Published in January number of the Journal.

Acinitis Prostatica, J. Henry Dowd, Buffalo. Published in December number of the JOURNAL.

Prostatectomy—Indications for Operation with remarks on the Technic of a Method of Operating, by Parker Syms, New York City.

DISCUSSION.

DR. J. HENRY DOWD of Buffalo: I was pleased to hear what Dr. Syms had to say as to the harm done in these cases by the use of steel catheters and similar instruments. Much damage is certainly done in this way. These instruments are poked through the prostate in all directions. I would make just one criticism on Dr. Syms's presentation of the subject—he made these operations seem too easy. I have done fifty or sixty of them, but when the ordinary physician gets into this region he is entirely in the dark. We used to be able to count the Buffalo physicians who did this work on our fingers, but now they are as numerous as the hairs of the head. I have in mind now one man who operated on one of these cases with the result that the water ran straight through into the rectum. I have gone in there twice since in an effort to repair the damage.

DR. W. B. JONES of Rochester: Water certainly does run down hill and for this reason, among others, I consider the perineal route the best. The rubber retractor of Dr. Syms is as far ahead of metal retractors as can be imagined, and with it injuries to the rectum are much less likely.

There have been fifty or sixty of these cases around here. Dr. Byam who has had this same operation, came here to tell us his experience, and he has read a paper before a neighboring county society, advising all suffering from similar conditions to secure relief in this operation of Dr. Syms.

DR. W. B. JOHNSON of Batavia: I would like to make one suggestion in regard to the rubber retractor—always test it before operating. I have had occasion to operate with one and the bulb came off.

DR. PARKER SYMS of New York City: I thank the doctor for suggesting that warning. Some of the earlier retractors were improperly made and the bulbs came off easily. And furthermore, rubber deteriorates with age, so that it is always wise to make sure before operating that the retractor is in good condition.

The following papers were then read: The Relation of Hyperchlorhydria to the Nestor Function of the Stomach, by Allen A. Jones, of Buffalo. Published in the January number of the JOURNAL.

Some Atypical Cases of Varicella, by A. Wood Ruggles of Rochester, N. Y.:

The afternoon session was called to order at 2.37 P. M., by the president Dr. W. B. Jones.

Under the head of Miscellaneous Business, Dr. Clarence A. Greenleaf, secretary, read a correspondence concerning a proposed amalgamation between the district branches of the State Society and the Medical Association of Central New York.

It was moved and seconded that the report of Dr. Greenleaf be received. Carried.

The correspondence follows:

Albany, N. Y., July 30, 1907.

DR. C. A. GREENLEAF, Canoga:

*Dear Doctor*—This is what lies in my mind regarding the relations between the State society organisations and existing voluntary societies covering a district of several counties. The contemplation of the former is to have in addition to the one general society, with its work including an annual meeting in Albany, a number of local societies which will be mainly for the purpose of providing for each a scientific and social meeting in the fall of the men of an area of a few counties. To this end the State is divided into eight districts. Their respective meetings will be conducted by themselves supplemented by the help and contribution of the general society. In this way the work of the State society will be brought nearer to the men of the State, many of whom can participate in its work through this branch society—scientifically, socially, or in whatever way they may desire—who could not reach often the general meeting in the winter. One after another of them will be made very likely a fuller semi-annual meeting of the whole society. Thus the whole medical profession is bound together in an organisation beginning with the county society, the larger district society and the executive body of the State society, membership in one being membership in all, and democratically open to every one.

But the district society is a new comer and has to make its place. It ought to make a good place for itself; but it ought not to compete with existing associations covering similar fields and having a similar purpose. I don't think it ought to crowd in and draw away from these established societies. Indeed, I don't think it can, for these societies, like yours and the association of northern New York, have too strong a hold on their membership. Besides, it is a question whether two bodies covering similar territory and holding fall meetings will exist side by side without detracting from each other. It would appear to me wise if they joined forces. Each has some good of its own; your society has its established clientele, history, and hold on the profession. Ours has its advantage in its relation to the State and county societies and being part of a wider organisation.

If some plan could be devised by which they could be combined and each contribute what it has to give to the union, without giving up anything on the part of either, it would be a good thing

I thought that perhaps you had printed copies of your organisation and constitution, and if so I would be glad to have one.

One difficulty has struck me as possible, viz: that the territory covered would disagree rather radically—that yours may take in parts of our fifth, sixth, and seventh districts. But I wish that my plan might appeal to your members for consideration, and that the wise men might see a way to effect a combination of resources, for it seems to me that variations of whatever sort might be adjusted, whether of territory, name or of organisation. Their ultimate purpose is almost identical.

Yours sincerely,

F. C. CURTIS.

Albany, N. Y., September 14, 1907.

DR. C. A. GREENLEAF,

Secretary, Med. Assoc. Central New York.

*Dear Doctor*—I thank you very much for the copy of by-laws, which I return. I have hoped for a union of the existing associations covering various parts of the State with our state society branch societies. I think this will be effected with the Medical Association of northern New York, which practically covers the same territory as our fourth branch society.

I see by your by-laws or constitution that your association takes in Onondaga and Oswego of our fifth, Chenango, Cortland, Madison and Chemung, of our sixth, all of our seventh except Steuben and all of our eighth. Thus it could hardly identify itself with any one.

Your body is a delegate body, with permanent members, from county societies, etc., with provision for a hearing from any member of a county society.

You cover too wide a territory to coalesce with one branch society, and are too strong to split up into all four and from what I gather of opinion of some of your members too old and established to give up to us.

It is in my mind desirable, though, that our societies should neither compete nor conflict with yours, and some sort of combination would be for the good of both—to avoid competition, to strengthen us new comers, and to ally yours in a degree with the state society, in the council which the district branch president is a member.

There is one proposition which appears to me easy to effect, involving little change in administration or method, and which will effect the main purpose, which no doubt will appeal to you as desirable. That is, to hold joint meetings. Your constitution provides for meetings in the various large cities. These are in our various districts. Buffalo is in our eighth district, Rochester in the seventh, Syracuse in the sixth, Auburn is also in the seventh, but if you had a membership sufficient in Elmira, that would be in our sixth. However, such a change is not important and perhaps not desirable, for Auburn is equally good, and three or four years no doubt elapse between it and Rochester as a place of meeting.

Having a common time and place of meeting, say at Syracuse, each body could hold its executive session apart and then join in the scientific meeting. The presidents of the two bodies could appoint a joint business committee. Each president could preside by arrangement at different sessions. I see no reason why, by a joint committee on nomination, the same man could not be made president for the year and likewise the vice-president; such committee could be made up before the meeting by the presidents, the officers nominated being from the districts where the meeting is to be next held. Then with a joint committee on scientific work a practical union of interest and resources could be effected.

Whether a closer union can be effected I do not know. Our branch societies make each its own by-laws subject to approval by the state society. Those affected could frame theirs to conform with this plan and I know no reason why they would not.

Yours very truly,
F. C. Curtis.

Dr. Wm. C. Krauss of Buffalo: I have been interested in this association of ours for a number of years, and others have seen it grow from small beginnings to its present size and influence. We have had large and enthusiastic meetings like this one, in Syracuse, Auburn and Buffalo. It seems to me that our territory is too wide for amalgamation, and that the *esprit de corps* is too good to justify us in giving up the individuality of the central New York and in allowing it to be split up into various district societies.

Dr. Henry Elsner of Syracuse: This question is one of vital importance and I agree entirely with the sentiments expressed by Dr. Krauss. This association should continue as a separate society. It should not die. It has been the idea of the district societies to discourage this sort of an organisation, but let us go on as before—and I am certain that the interest will not die out, and that this association will continue to exist and do good work in the future as it has in the past.

Dr. Snow of Batavia: I agree with what has been said by Drs. Krauss and Elsner. I have the honor of being vice-president of the eighth district branch, which includes counties all but three of which are included in this association, and I think that our organisation as it exists today is the better arrangement.

At the suggestion of the president, Dr. W. B. Jones, a motion was made and seconded, that a committee be appointed to draft a reply to these overtures for amalgamation. Carried. The committee, subsequently announced by the president, consisted of Drs. Young, Miller and Johnson.

Report of committee as follows:

Your committee relative to the communication of the state society concerning the amalgamation of this association with the district branch of the state society, would respectfully report:

That observation indicates that it is the desire of the members of this organisation that it remain distinct and independent of the district organisation of the state society, believing that such union would in no way be of any advantage to the medical profession of central and western New York. Indefinite postponement of this subject is recommended.

It is further recommended that the secretary of this association be authorised to so report to the state society the concensus of opinion of the members of this association.

A. A. Young,

H. B. Miller,

Wm. D. Johnson.

The report of the committee on credentials was received and accepted. It follows:

The Committee on Credentials recommend the election to membership of H. J. Knickerbocker, M. D., Geneva, N. Y. On examination we find he was duly recorded as a delegate from the Ontario County Medical Society, for the requisite number of meetings.

Eugene A. Smith, Chairman.

The election of officers for the ensuing year resulted as follows: President, C. C. Frederick, M. D., Buffalo; first vice-president, M. P. Conway, M. D., Auburn; second vice-president, A. A. Young, M. D., Newark. The meeting adjourned at 5.30 P. M., to meet in Buffalo, Tuesday, October 20, 1908.

